# Expression of concern: Copper nanoparticle anchored biguanidine-modified Zr-UiO-66 MOFs: a competent heterogeneous and reusable nanocatalyst in Buchwald–Hartwig and Ullmann type coupling reactions

**DOI:** 10.1039/d4ra90111h

**Published:** 2024-09-27

**Authors:** Hojat Veisi, Narges Neyestani, Mozhgan Pirhayati, Sheida Ahany Kamangar, Shahram Lotfi, Taiebeh Tamoradi, Bikash Karmakar

**Affiliations:** a Department of Chemistry, Payame Noor University (PNU) Tehran Iran hojatveisi@yahoo.com; b Department of Applied Chemistry, Faculty of Science, Malayer University Malayer Iran; c Chemistry Department, Production Technology Research Institute-ACECR Ahvaz Iran t.tabss@yahoo.com; d Department of Chemistry, Gobardanga Hindu College 24-Parganas (North) India bkarmakar@ghcollege.ac.in

## Abstract

Expression of concern for ‘Copper nanoparticle anchored biguanidine-modified Zr-UiO-66 MOFs: a competent heterogeneous and reusable nanocatalyst in Buchwald–Hartwig and Ullmann type coupling reactions’ by Hojat Veisi *et al.*, *RSC Adv.*, 2021, **11**, 22278–22286, https://doi.org/10.1039/D1RA02634H.

*RSC Advances* is publishing this expression of concern in order to alert readers that concerns have been raised over the integrity of the data published in this article. The authors have been contacted but have not responded to requests to provide raw data. An expression of concern will continue to be associated with the article until a conclusive outcome is reached.

Laura Fisher

16th September 2024

Executive Editor, *RSC Advances*

